# Foreign investments and load capacity factor in BRICS: the moderating role of environmental policy stringency

**DOI:** 10.1007/s11356-023-31814-9

**Published:** 2024-01-13

**Authors:** Metin Yıldırım, Mehmet Akif Destek, Müge Manga

**Affiliations:** 1 Department of International Trade and Finance, Necmettin Erbakan University, Konya, Turkey; 2 Department of Economics, Gaziantep University, Gaziantep, Turkey; 3 Adnan Kassar School of Business, Lebanese American University, Beirut, Lebanon; 4 Department of Economics, Faculty of Economics and Administrative Sciences, Erzincan Binali Yıldırım University, Erzincan, Turkey; 5 UNEC Research Methods Application Center, Azerbaijan State University of Economics (UNEC), Baku, Azerbaijan

**Keywords:** Foreign direct investments, Load capacity factor, Environmental stringency, Renewable energy, BRICS

## Abstract

This research examines whether environmental regulations have a moderating effect on the link between foreign direct investment and the environment, as well as the effect of foreign capital investments on environmental quality for BRICS nations. In this approach, using second-generation panel data methodologies for the period 1992–2020, the impacts of foreign direct investments, real national income, consumption of renewable energy, and environmental stringency index on the load capacity factor are explored in the base empirical model. In order to test if there is any evidence of a potential parabolic link between economic growth and environmental quality, the model also includes the square of real national income. In addition, in the robustness model, the moderating role of environmental policy on foreign investment and environmental quality is checked. Empirical results show a U-shaped association between environmental quality and economic development. The usage of renewable energy and the environmental stringency index is also shown to improve environmental quality, although foreign direct investments decrease it. Finally, it is determined that environmental regulations are effective in undoing the negative impacts of foreign capital investments on environmental quality, demonstrating the validity of their moderating function.

## Introduction

The strategies for addressing climate change play a critical role in the solution-focused approach to the primary goals established to ensure sustainable development. While climate change adaptation lessens the susceptibility to global climate variability, it also increases the capacity to anticipate and adapt to future climatic changes (Abeygunawardena et al. 2004). Regardless of wealth disparities between nations, adherence to development objectives is crucial for achieving sustainable development on a global scale. Therefore, inclusive policy methods that include poor nations in the problem-solving process are required for the achievement of sustainable development (UN 2008). The battle against stress and climate change, however, has several challenges in poor nations (Bhattacharya et al. 2023). Although developing nations are the ones most vulnerable to environmental issues in contemporary society, these nations do not create pollution control measures and are unable to offer enough implementation mechanisms to guarantee the effectiveness of pertinent laws (JICA 2005).

The shortage of funds in emerging nations is one of the key causes of this predicament. Foreign direct investments are permitted in developing nations primarily to address the capital shortage and address numerous macroeconomic issues (Nunnenkamp 2001; Wang and Chen 2014). Foreign investors entering the country, however, typically only care about their short-term profitability, oblivious to the high upfront costs, protracted payback periods, and confrontation with significant risks associated with investments in projects that can reduce greenhouse gas emissions through renewable resources, energy efficiency, and afforestation (Beg et al. 2002). Since the 1970s, this circumstance has prompted consideration of the link between foreign direct investments and environmental contamination (Gill et al. 2018).

With the implementation of the North American Free Trade Agreement (NAFTA), concerns about the transfer of polluting industries to developing nations because of the liberalization of capital circulation start to get more serious. The agreement expressed disapproval of the clear disparities in the expenses of eradicating environmental issues between the USA and Mexico as well as the escalating environmental issues in the Maquiladora area of Mexico. As polluting businesses move into Mexico, the pact is expected to be a catastrophe for the environment there (Ederington 2007). This results in the topic being addressed in intellectual, political, and industrial aspects because of the growing discourse on the link between foreign investments and the environment. At that time, a range of viewpoints about the relationship between foreign investments and the environment arose. The pollution haven hypothesis (PHH) is one of them. According to Gill et al. (2018), this theory was put out by Copeland and Taylor in 1994 and is based on the variance in how harsh environmental pollution laws are in different nations. In other words, according to this theory, certain industries with high levels of pollution and consumption will move from developed to developing nations due to lax environmental regulations through trade and foreign direct investment, leading to a significant increase in pollutant emissions in the host nations (Apergis et al. 2023).

In the framework of the North–South link, Copeland and Taylor (1994) addressed the connection between commerce and the environment. The research makes the assumption that technology (or human capital) is different in the North and the South. Since the North has a greater wealth level than the South, internal environmental policy is used to define a harsher environmental policy. The northern area, which specializes in the development of environmentally friendly products, has a competitive advantage based on revenue. Due to its lower salaries and laxer environmental regulations, the South—which is a continuous recipient of foreign direct investment—is a net exporter of commodities that are dirty. Therefore, the pollution haven hypothesis (PHH) is shown by the labor-rich Southern Region (Bogmans and Withagen 2010).

Environmental regulations, on the other hand, are a well-known key element in the battle against climate change. Environmental regulation, in general, refers to the pertinent policies and actions taken by governments to limit the production and operational activities of businesses and decrease pollution (Wang et al. 2022). The tools at the disposal of governments for implementing environmental policy are many. These consist of legislative (or “command and control”) tools, market-based tools (such as levies and tradeable permits), subsidies, environmental management programs, and communication campaigns. Since they have a direct influence on environmental protection and are straightforward to execute, tax (and tradable permits) approaches are primarily used by global economies (OECD 2010). The key question in this situation is whether a moderating element exists that will reduce or completely remove any potential environmental harm that comes from foreign investments in developing nations.

When determining whether environmental legislation can undo the damaging consequences of foreign direct investment on environmental deterioration, two key concerns come into play. The initial act is to choose the indication indicating environmental regulations that is the most accurate. There have recently been more studies on the impact of the environmental policy stringency index (EPSI) on climate change, as seen in studies like Li et al. (2022b), Kruse et al. (2022), Wen et al. (2022), Wang et al. (2022), Hassan et al. (2022), Udeagha and Ngepah (2023), Tariq and Xu (2022), and Yirong (2022). As stated by Chen et al. (2022), the index is based on the execution of different policies based on the market, non-market, and technology. EPSI combines different environmental policy indicators, including those for energy and transportation (Botta and Koźluk 2014). It is concentrated on air and climate policies in areas of primary environmental importance, particularly EPSI, environmental taxes, support for renewable energy and energy efficiency (tariff guarantee, renewable energy certificates, R&D expenditures), performance standards (emissions for coal-fired power plants) limit values, and limits of sulfur content in diesel fuels (OECD 2016). The key benefit of using EPSI as a significant indication of environmental restrictions is that it is done for pollutants that are significant to markets and are therefore applied to substantially similar pollutants, based on the application of policies. Comparing the stringency of the index’s covered policy instruments is not too difficult. Since it covers more countries and more time than previous indicators, the final indicator is simple to update and grow (OECD 2016).

The second important issue is which indicator to look for to represent environmental degradation. In recent years, the carbon emission parameter is primarily used as an environmental pollution indicator by Adeleye et al. (2023), Wang et al (2023), Ferdous and Ahmed (2022), Tao et al. (2023), Destek et al. (2023a), and Zeng et al. (2023). However, carbon emissions are not an appropriate criterion to be used alone to quantify environmental sustainability, as has recently been noted (Solarin 2019), since they do not represent the whole effect of human activities on the environment. The indicator of carbon emissions does not consider factors that contribute to environmental pollution, such as the eradication of forests and productive agricultural areas, the contamination of freshwater supplies, and the extinction of biological variety. Since carbon emissions are insufficient as an indication of environmental contamination, a more comprehensive metric is required (Aydın, 2020). In research for this aim, it is clear that the ecological footprint metric has recently received increased attention (Kongbuamai et al. 2020; Destek 2019; Ahmed et al. 2020; Li et al. 2022a; Ansari 2022; Appiah et al. 2023; Javed et al. 2023; Destek et al. 2023b and Saqib et al. 2023). According to Wiedmann and Barrett (2010), ecological footprint is a factor that explains why people need biological resources on a global scale. The demand for nature as well as the capacity of nature to meet the demand (supply-side environmental concerns) are not taken into account in environmental approaches, which is an important criticism of the ecological footprint (Pata and Isik 2021; Ullah et al. 2023; Awosusi et al. 2022). Moreover, it can be claimed that the load capacity factor has a number of advantages over the ecological footprint in terms of illustrating environmental quality since it is challenging to examine the variables that affect how intensively the items produced in each sector are utilized (Siche et al. 2010). According to Siche et al. (2010), the load capacity factor describes a region’s ability to utilize its natural resources in a sustainable manner. This element is crucial for maintaining the ecosystem’s balance. The ecological footprint and the load capacity factor are contrasted to track the ecosystem’s equilibrium. When a region’s ecological footprint exceeds its biocapacity, this indicates that the region’s natural resources are being exploited and that consumption is not sustainable (Dam et al. 2023). With the aid of the threshold value, it is possible to deduce that a load capacity factor of ≥ 1 indicates that the state of the environment is sustainable while a value of ≤ 1 indicates that environmental deterioration is not (Adebayo et al. 2022). In lieu of the traditional EKC hypothesis, the load capacity factor (LCF), which is an environmental quality indicator, is also investigated in research based on the load capacity curve (LCC). This theory, which takes into account the potential U-shaped link between economic growth and environmental quality, states that environmental quality first declines as income rises and then gradually increases until national income reaches a particular level (Yang et al. 2023; Pata and Destek 2023; Caglar et al. 2023; Pata et al. 2023; Kartal and Pata 2023; Jin et al. 2023; Pata et al. 2024).

The purpose of this study is to ascertain, in light of the aforementioned discussions, whether more stringent environmental regulations in BRICS (Brazil, Russia, India, China, and South Africa) nations can undo the negative effects of foreign investments on environmental quality. There are a few justifications for selecting the BRICS nations as research samples. First of all, these nations have difficulty reaching the targets established in regard to sustainable development objectives. In order to continue economic development and raise their technology, management abilities, capital, and living standards in different ways, the BRICS nations pursue an international trade strategy that is welcoming of foreign direct investments (Tan and Uprasen 2022). The yearly foreign direct investment inflows of the BRICS have surged more than four times, according to the UNCTAD (2023) study. From $84 billion in 2001 to $355 billion in 2021, FDI inflows rose. From 11% in 2001 to 22% in 2021, their proportion of FDI inflows into the world more than quadrupled (UNCTAD 2023). On the other hand, the BRICS group’s environmental impact and load-bearing capacity are continually growing as a result of the rising foreign direct investments. China and India are among the nations with the largest disparities in ecological footprint and load-carrying capability globally, according to statistics from the World Population Review (2023). The BRICS nation group’s environmental quality is suffering as a result of this predicament. According to the Global Footprint Network (2022), the per capita load-bearing capacity in 2022 is as follows: India 0.4, China 0.8, South Africa 1.2, Russia 7.5, and Brazil 8.3 hectares. And it is obvious that South Africa faces a threat to the environment’s sustainability. When we look at the results from empirical analysis, the findings suggest that the detrimental impacts of foreign direct investment on the environment are reversed by the tightening of environmental rules. In other words, environmental restrictions’ ability to moderate behavior has been established.

The following are some possible contributions that the research might make to the literature: (i) The research is the first to use the load capacity factor to examine the validity of the pollution haven hypothesis for the BRICS nations. (ii) The research will provide more reliable findings on the environmental effectiveness of environmental laws in BRICS countries if it uses the recently created environmental stringency index as an indicator of environmental regulations. (iii) Due to the empirical analysis’s use of second-generation panel data methodologies, potential shock transfers across BRICS nations are also taken into consideration. (iv) Rather than focusing on the direct consequences of foreign direct investments on the environment, it is being explored whether environmental rules will be able to mitigate any potential detrimental effects. Since this kind of research does not exist in the literature, it is anticipated that major contributions will be produced. (v) The validity of the load capacity curve hypothesis, which has been newly introduced to environmental economics, is also being investigated.

The rest of this paper is organized as follows: In “Literature review” section, the results of the extant literature are evaluated critically, and the gaps in the literature are identified. The econometric techniques utilized to run the various empirical models are outlined in Part 3; the results are summarized and explained in Phase 4; and the empirical project is concluded with policy recommendations and closing remarks in “Conclusions and policy implications” section.

## Literature review

In the literature concerning sustainable development, numerous parameters representing environmental degradation and/or quality in developing countries are observed. The primary objective of this study is to estimate the relationship between foreign direct investments (FDI) affecting environmental quality and environmental regulations for BRICS countries. Therefore, this section is presented under two headings. The first section discusses the relationship between foreign direct investments and environment, while the second section addresses studies focusing on the relationship between environmental regulations and environment.

### Foreign direct investment and environment nexus

In recent literature, many studies focus on the role of foreign direct investment (FDI) in influencing environmental pollution, considering it as one of the main channels of technology transfer and its impact on the economic structures of recipient countries (Udemba 2020). There is a divergence of opinions in the literature regarding the effect of FDI, an important factor that can have different effects on environmental quality (Saqib et al. 2023).

The relationship between foreign direct investment (FDI) and environmental pollution is examined by Grossman and Krueger (1991) within the framework of three effects. The first of these is the scale effect. In the scale effect, the pollution haven hypothesis emerges, suggesting that FDI has positive effects on the economy but negative effects on the environment. In the composition effect stage, FDI influences the country's industrial composition and is a phase that enables the development of ecologically more destructive or less polluting industries. The environmental impact of this process can be either positive or negative. Finally, the technology effect is the stage where FDI creates positive effects on the environment through the transfer of green technology. In this stage, while the ecological footprint decreases, there is also the potential for new developments that can increase economic growth. In this stage, the pollution halo hypothesis, indicating a negative relationship between foreign direct investment and environmental pollution, is valid.

Studies accepting the pollution haven hypothesis state that in countries hosting foreign direct investments, environmental concerns are overlooked, leading to an increase in environmental pollution as investments increase. In these studies, Chowdhury et al. (2021) found through a panel quantile regression model for the period 2002–2016 in 92 countries that foreign direct investments increased the ecological footprint, which is an important indicator of environmental pollution. Baloch et al. (2019) also obtained results indicating an increase in the ecological footprint after foreign direct investments for 59 Belt and Road countries in the period 1990–2016, using the Driscoll-Kraay panel regression model. In addition, Gorus and Aslan (2019) found evidence that with the increase in foreign direct investments entering the country, environmental quality decreased; in other words, the ecological footprint increased, for MENA countries. Similar results are obtained for ASEAN countries by Zhu et al. (2016), for Nigeria by Dada and Akinlo  (2021), for 119 developed and developing countries by Uddin et al. (2023), for PIIGS nations by Balsalobre-Lorente et al. (2022), and for G-20 countries by Musah et al. (2022).

Moreover, some studies argue that as foreign direct investments (FDI) increase, technology transfer leads to a decrease in environmental pollution, or in other words, they support the pollution halo hypothesis. In these studies, Deng and Xu (2015) used the spatial error model (SEM) and the spatial lag model (SLM) methods for the years 2000 and 2010, covering 200 selected countries. They indicated that foreign direct investment had a positive impact on the host country’s environment, benefiting from scale and technical effects. Udemba (2020) employed the NARDL method for India and estimated a negative relationship between ecological footprint and foreign direct investments. Similarly, Udemba (2021) found a similar result for the UAE for the period 1990–2018 using the ARDL method. Chaouachi and Balsalobre-Lorente (2022) used the same method for Algeria for the years 1975–2014 and found that foreign direct investments encouraged the country to adopt cleaner energy usage, thereby reducing the negative environmental impact of fossil energy consumption. Saqib et al. (2023) used the CS-ARDL test for 16 European countries for the period 1990–2020, and it is concluded that foreign direct investments led to a decrease in ecological footprint. Roy (2023) employed a structural break unit root test for India for the years 1990–2016 and found that foreign direct investments resulted in a decrease in ecological footprint.

In recent times, there have been some studies in the existing literature that focus on the relationship between foreign direct investments and an important indicator of environmental quality, which is the load capacity, albeit to a lesser extent. For instance, Adebayo et al. (2022) found, for the period 1975q1–2018q4 in Thailand, that the pollution haven hypothesis is valid in the relationship between foreign direct investment and environmental quality represented by load capacity; in other words, foreign direct investments reduce load capacity (reduced environmental quality). Similarly, Soto (2023) obtained a similar result for Spain during the period 1961–2018.

### Environmental regulations and environment nexus

Environmental regulations encompass regulations that can contribute to improving environmental quality, ensuring long-term sustainability, and promoting green technology (Wolde-Rufael and Weldemeskelb 2020). Therefore, in general, it is expected that implemented environmental regulations will lead to improvements in environmental quality indicators.

From studies examining the relationship between the environmental policy stringency index, one of the environmental regulation indicators, and environmental pollution, Yirong (2022) used a panel ARDL method and a non-linear model to find that carbon emissions decreased for China, USA, India, Russia, and Japan. Furthermore, Ahmed and Wang (2019) probed the relationship between carbon emissions and environmental stringency index for China during the period of 1990–2012. The findings indicate that an increase in the environmental stringency index has a negative impact on carbon emissions, leading to a reduction in environmental pollution. A similar result is estimated for 32 countries during the period of 1990–2015 from Albulescu et al. (2022). The results show that increasing environmental policy stringency has a negative effect on emissions and that environmental stringency has a stronger impact in countries with lower carbon emission levels. Ahmed (2020) found that increasing environmental policy stringency led to a decrease in carbon emissions by promoting the use of green technology in 20 OECD countries. Chen et al. (2022) analyzed the relationship between the environmental stringency index and various environmental pollution indicators in China during the period of 1993–2019. Using the NARDL method, the long-term findings showed that positive shocks in environmental policy stringency reduced carbon emissions by 0.94% and greenhouse gas emissions by 0.77%, while negative shocks in environmental policy stringency increased N2O emissions by 0.17%, PM2.5 emissions by 0.50%, and carbon emissions by 0.63%. Udeagha and Ngepah (2023) analyzed the relationship between carbon emissions, an indicator of environmental pollution, and the environmental stringency index for BRICS countries during the period of 1960–2020. Findings obtained using the CS-ARDL method indicated that an increase in the index reduced carbon emissions. Similar results were also reported in the following studies: Kongbuamai et al. (2021) for BRICS countries; Umar and Safi (2023) for OECD countries; Fatima et al. (2023) for 36 OECD countries.

In the study by Daniel Balsalobre-Lorente et al. (2023) which examines the relationship between the ecological footprint, another important indicator of environmental pollution, and the environmental stringency index, it was found that for APEC countries during the period of 1994–2018, using second-generation panel estimation methods, the environmental stringency index reduced the ecological footprint. A similar relationship was investigated by Kongbuamai et al. (2021) for BRICS countries. Using the DSUR method and the Dumitrescu and Hurlin panel causality tests, the analysis concluded that an increasing environmental stringency index reduced the ecological footprint. The relationship between these two parameters was also estimated by Chu and Tran (2022) for the period of 1990–2015 in 27 OECD countries. Using panel quantile regression, it is stated that increasing the stringency index reduced the ecological footprint. Similar results are reported in the following studies: Appiah et al. (2023) for OECD countries; Kruse et al. (2022) for OECD countries; Assamoi and Wang (2023) for China and US countries.

## Empirical methodology

### Model and data

The following is the panel version of the empirical model developed in the study to examine how foreign investments affect environmental quality:

1 $${lc}_{it}={a}_{0}+{a}_{1}{gdp}_{it}+{a}_{2}{gdp}_{it}^{2}+{a}_{3}{ren}_{it}+{a}_{4}{fdi}_{it}+{a}_{5}{esi}_{it}+{u}_{it}$$

where “lc” stands for load capacity, which represents environmental quality; “gdp” is real gross domestic product, which stands for economic growth; “ren” is renewable energy and represents renewable energy consumption; “fdi” is foreign direct investments and is used as a proxy for foreign investments; and “esi” is used as environmental stringency index, which stands for environmental regulations. Additionally, “gdp^2^” is incorporated into the model to look for a potential parabolic relationship between environmental quality and economic growth. In empirical analysis, this model is referred to as Model I.

According to the pollution haven hypothesis, which is supported by the empirical literature, it is predicted that foreign investments will lead to a decline in the environmental quality in emerging nations. Based on this, the second empirical model developed to determine how the expansion of environmental regulations in developing countries, in addition to their direct effects on the environment, transforms the environmental effects of foreign investments is as follows:

2 $${lc}_{it}={\beta }_{0}+{\beta }_{1}{gdp}_{it}+{\beta }_{2}{gdp}_{it}^{2}+{\beta }_{3}{ren}_{it}+{\beta }_{4}{fdi}_{it}+{\beta }_{5}{esi}_{it}+{\beta }_{6}{esi*fdi}_{it}+{\varepsilon }_{it}$$

where “esi*fdi” is the interaction term added to the model to track the combined impact of foreign investments and the environmental tightness index. The parameter of this variable reveals the impact of rising environmental legislation and foreign direct investment on environmental quality. From a different angle, this parameter illustrates how foreign direct investments affect environmental quality in nations with the highest levels of environmental regulation among those shown in the panel. In empirical analysis, this model is referred to as Model II.

The analysis uses annual data for the BRICS (Brazil, Russia, India, China, and South Africa) nations for the years from 1992 to 2020. Table 1 lists the data’s sources as well as the variables’ measurement units. We utilize the natural logarithmic form of each variable.

**Table 1:** Definitions and sources of variables

Variables	Symbols	Definitions	Sources
Environmental quality	lc	Biocapacity per capita/ecological footprint per capita	Global Footprint Network (2022)
Economic growth	gdp	GDP per capita (constant 2015 US$)	World Bank (2022)
Renewable energy	ren	Renewable energy consumption (% of total energy consumption)	World Bank (2022)
Foreign direct investment	fdi	Foreign direct investment, net inflows (% of GDP)	World Bank (2022)
Environmental regulations	esi	Environmental stringency index	OECD (2022)

Table 2 includes descriptive statistics that were computed to observe the properties of the variables. As a result, it can be observed that Brazil had the greatest load capacity level (4431) in 1992 and China had the lowest load capacity level (0.227) in 2020. China will also have the greatest per capita income in 2020, with 10,358 USD. India attained the greatest value in terms of the proportion of renewable energy consumption in overall energy consumption in 1993 with a level of 51.49%. China received 6.187% of all inflows of foreign direct investment (measured as a percentage of gross domestic product) in 1993. Furthermore, China stands out in 2020 as having the strictest environmental rules, with an index score of 3139. China is also the country with the highest environmental standards. All the variables—aside from “gdp”—are positively skewed when we examine the skewness-kurtosis forms of the variables, whereas only “gdp” is negatively skewed. Additionally, whereas “gdp,” “ren,” and “fdi” exhibit leptokurtic distribution, “lc” and “esi” exhibit platykurtic distribution.

**Table 2:** Descriptive statistics

	lc	gdp	ren	fdi	esi
Mean	1.179	5254.541	24.611	2.111	0.884
Median	0.465	5798.915	18.570	1.842	0.694
Maximum	4.431	10358.170	51.490	6.187	3.139
Minimum	0.227	546.441	3.180	0.002	0.010
Std. Dev	1.215	2923.326	17.221	1.455	0.764
Skewness	1.304	-0.167	0.153	0.553	1.333
Kurtosis	3.186	1.830	1.407	2.427	4.058
Observations	145	145	145	145	145

### Methodology

#### Preliminary tests

To determine if there is a strong correlation between independent variables and potential multicollinearity in the created empirical model, correlation analysis is used in the first stage of empirical analysis. Then, it is necessary to regulate cross-section dependence (CSD), a recent development that has become more significant, particularly in light of the globalization phenomena. The cross-sectional dependency (CD) test created by Pesaran (2004) is used in this approach. The stationarity processes of the variables should be checked using a panel unit root test that enables cross-sectional dependence after analyzing the cross-sectional dependence (the anticipated condition is that the cross-sectional dependence is legitimate). Here, Pesaran’s (2007) CIPS unit root test is applied. In order to determine if a long-term connection between the variables is legitimate, the panel cointegration test should be utilized. The panel cointegration test, which allows for inter-country reliance and is based on the error correction model created by Westerlund (2007), is used to examine the cointegration connection.

#### CS-ARDL estimation technique

The CS-ARDL approach is used in the research to examine the short- and long-term impacts of real national income, renewable energy consumption, foreign direct investments, and the environmental tightness index on the load capacity factor. The 1-year lag of the regression variable is seen by the CS-ARDL paradigm as a weak exogenous regressor during the error correction procedure. According to Chudik and Pesaran (2015), the CS-ARDL approach enhances the ARDL model by accounting for cross-sectional dependency in the error term by a linear combination of the mean cross sections of both dependent and independent variables:

3 $${EQ}_{i,t}={\beta }_{i}+{\mu }_{ji}\sum_{j=1}^{ay}{EQ}_{i,t-j}+{\alpha }_{ji}\sum_{j=0}^{bx}{X}_{i,t-j}+{\mu }_{ji}\sum_{j=0}^{c}\overline{{EQ }_{i,t-j}}+{\alpha }_{ji}\sum_{j=0}^{d}\overline{{X }_{i,t-j}}+{e}_{i,t}$$

where EQ is the load capacity factor which indicates environmental quality and $ay$ ve $bx$ are optimum lag lengths; ${X}_{i,t}$ is the regressor matrix that combines gdp, ren, fdi, and esi. In addition, the long-run parameters are computed as follows:

4 $${\widehat{\theta }}_{CS-ARDL,i}=\frac{\sum_{j=0}^{bx}{\widehat{\alpha }}_{ji}}{\sum_{j=1}^{ay}{\widehat{\mu }}_{ji}}$$

The following describes the model’s adaptation to the error correction model:

5 $$\begin{array}{c}\Delta {EQ}_{i,t}={\beta }_{i}+{\varnothing }_{i}\left({EQ}_{i,t-1}-{{\omega }_{i}X}_{i,t-1}\right)+{{\mu }_{ji}}^{*}\sum_{j=1}^{ay-1}{\Delta EQ}_{i,t-j}+{{\alpha }_{ji}}^{*}\sum_{j=0}^{bx}\Delta {X}_{i,t-j}+\\ {\gamma }_{i}\sum_{j=1}^{c}\overline{{EQ }_{i,t-j}}+{v}_{i}\sum_{j=0}^{d}\overline{{X }_{i,t-j}}+{\rho }_{i}\sum_{j=1}^{c-1}\overline{{EQ }_{i,t-j}}+\\ {\sigma }_{i}\sum_{j=0}^{d-1}\overline{{X }_{i,t-j}}{e}_{i,t}\end{array}$$

where ${\varnothing }_{i}$=-(1-$\sum_{j=1}^{ay}{\mu }_{ij})$ is the error correction term and $\Delta$ is the first difference operator.

## Empirical results and discussions

### Empirical findings

It is a well-known concern that certain issues (multicollinearity, cross-sectional dependence, and unit root) may cause the results from panel data analyses to be erroneous. In order to prevent a potential multicollinearity issue, the correlation relationships between the variables are first examined in this manner and are shown in Table 3. Therefore, although there are negative correlations in the lc-ren and lc-esi interactions, positive correlations are true in the lc-gdp and lc-fdi relationships. Additionally, it is possible to claim that the multicollinearity issue is invalid since a significant correlation between independent variables is not legitimate.

**Table 3:** Correlations among variables

	lc	gdp	ren	fdi	esi
lc	1				
gdp	0.446725	1			
ren	− 0.099786	− 0.497836	1		
fdi	0.121523	0.12555	0.167694	1	
esi	− 0.479813	− 0.073721	0.004723	− 0.117932	1

Cross-sectional dependency should be examined as the second crucial problem. This scenario relates to whether or not the nations in the panel are dependent on the same factors. Results of the CD test devised by Pesaran (2004), which was used to verify cross-sectional dependency in Table 4, show that other BRICS nations are likewise impacted by a shock in any of the model’s variables when the same variable is also affected.

**Table 4:** Results from the CSD test

	CD-statistics	*p*-value
lc	7.970***	0.000
gdp	16.000***	0.000
ren	8.920***	0.000
fdi	13.010***	0.000
esi	11.340***	0.000

^*^, **, and *** indicate statistical significance at 10, 5, and 1% levels, respectively

The stationarity processes of the variables are evaluated in the next step because analyses using non-stationary series run the risk of false regression. The CIPS unit root test, developed by Pesaran (2007) and used for this purpose, allows for cross-sectional dependency, and the findings show that the null hypothesis suggesting a unit root cannot be ruled out in the level forms of the variables (Table 5). On the other hand, it can be observed that the variables are stationary, and the null hypothesis is rejected in the first difference forms for all variables.

**Table 5:** Results from panel unit root test

Variables	Level	First differences
lc	− 2.575	− 4.207***
gdp	− 1.498	− 2.898**
ren	− 2.439	− 3.613***
fdi	− 2.564	− 5.183***
esi	− 2.713	− 5.089***

^*^, **, and *** indicate statistical significance at 10, 5, and 1% levels, respectively. Critical values for 10, 5, and 1% are − 2.73, − 2.86, and − 3.10, respectively

The validity of a long-term link is shown by the fact that the variables are integrated in the first order, which suggests a potential cointegration relationship between the variables. The panel ECM-based cointegration test of Westerlund (2007) is used in this situation, and the results are shown in Table 6. The null hypothesis asserting that cointegration is not valid by four distinct statistics is rejected when the panel cointegration test results for Model I, which account for intercountry reliance, are analyzed. As a result, the variables “gdp,” “ren,” “fdi,” and “esi” are cointegrated. On the other hand, group-tau, panel-tau, and panel alpha statistics strongly reject the null hypothesis in light of the findings of the cointegration test conducted within the context of Model II. This result shows the cointegration of “gdp,” “ren,” “fdi,” “esi,” and “es*fdi.” In this situation, second-generation cointegration estimators need to be used to examine the long-term impacts of independent variables on the load capacity for both models.

**Table 6:** Results from the panel cointegration test

Statistics	Model I	Model II
Gt	− 4.163*** [0.000]	− 3.097** [0.022]
Ga	− 22.397*** [0.000]	− 15.471 [0.128]
Pt	− 9.468*** [0.000]	− 6.354** [0.031]
Pa	− 25.862*** [0.000]	− 16.859*** [0.002]

^*^, **, and *** indicate statistical significance at 10, 5, and 1% levels, respectively. Numbers in brackets are *p*-values

Table 7 provides the findings of the CS-ARDL test, which is used to distinguish between the short- and long-term impacts of the independent variables and takes the CSD problem into consideration. The long-term results obtained for Model I show that although the parameter of the square of real income is positive, the coefficient of real income is negative. This research suggests that there is a legitimate U-shaped association between environmental quality and economic development. In other words, starting at a specific real income level (a turning point), environmental quality begins to rise while load capacity declines in the early phases of economic expansion. This result is consistent with the Kuznets curve environmental theory based on environmental deterioration. On the other hand, the load capacity factor rises as the proportion of renewable energy in the energy mix of the BRICS nations grows. In reality, the load capacity factor rises by 0.10% over time for 1% increase in the amount of renewable energy. On the other hand, it is determined that the rise in foreign investment has a detrimental impact on the environment. Technically, a 1% increase in FDI would result in a 0.01% decrease in load capacity. This result demonstrates that the pollution haven theory applies to the BRICS nations. The load capacity factor rises by 0.01% for 1% increase in the environmental stringency index when we concentrate on the efficiency of environmental laws. In other words, environmental rules are a powerful instrument employed in the BRICS nations to improve environmental quality.

**Table 7:** Results from CS-ARDL estimation

	Model I	Model II
Short-run results		
gdp	− 1.674*** [0.562]	− 2.936*** [0.523]
gdp^2^	0.105** [0.056]	0.274*** [0.078]
ren	0.210* [0.121]	0.206* [0.120]
fdi	− 0.006** [0.002]	− 0.011*** [0.003]
esi	0.030*** [0.009]	0.023* [0.013]
esi*fdi	−	0.003 [0.002]
ECT(− 1)	− 0.801*** [0.047]	− 0.644*** [0.055]
Long-run results		
gdp	− 0.907** [0.442]	− 1.703*** [0.128]
gdp^2^	0.056*** [0.202]	0.108*** [0.012]
ren	0.101* [0.056]	0.102* [0.055]
fdi	− 0.003** [0.001]	− 0.005*** [0.001]
esi	0.015*** [0.004]	0.017*** [0.007]
esi*fdi	-	0.004*** [0.001]
*R*^2^	0.450	0.520

^*^, **, and *** indicate statistical significance at 10, 5, and 1% levels, respectively. Numbers in brackets are standard errors

Regarding the long-term results for Model II, it seems that, like the other model, the U-shaped link between economic performance and environmental quality is also supported. In actuality, the parameter of real income is negative whereas the parameter of real income squared is positive. In a similar vein, environmental restrictions and growing use of renewable energy both improve the environment. Additionally, the load capacity factor for the BRICS nations decreases due to the growth in foreign direct investment inflow. In order to reduce or completely remove the negative impact in question, the interaction term (esi*fdi) was included in the model. It is determined that this interaction term was the center of our attention and that the parameter of this variable has a positive sign and is statistically significant. As a result, ecologically hazardous investment attempts by foreign money may be stopped when environmental rules rise in the BRICS nations. In other words, among the BRICS nations, direct foreign investment inflows benefit countries with strict environmental regulations in terms of environmental quality. This result confirms that environmental restrictions have a moderating effect on the link between foreign investments and the environment.

Regarding the short-term results, both models demonstrate the U-shaped link between economic development and environmental quality. In actuality, real income’s parameter is negative while its square’s parameter is positive. Similar to the long term, environmental quality is favorably impacted by rising renewable energy use and environmental legislation. Foreign direct investment’s short-term coefficient is likewise unfavorable and statistically significant. Contrary to long-term results, the parameter of the “esi*fdi” interaction term is statistically negligible; hence, the moderating effect of environmental restrictions is invalid in the short run. This circumstance demonstrates that, in the near term, environmental rules are unable to undo the damaging environmental consequences of foreign direct investments. Furthermore, the ECM coefficient indicates an 80% and 64% annual adjustment to reach long-run equilibrium, and it is statistically significant at the 1% significance level.

### Robustness check

The CCE-MG estimator created by Pesaran (2006) is used to assess the robustness of the results acquired in the previous step, and the results are provided in Table 8. The test’s findings validate the U-shaped association between economic development and environmental quality. It looks at long-term impacts between cointegrated variables and may be utilized in the presence of CSD. Real income has a negative long-term coefficient for both models, but the square of real income has a positive long-term coefficient. In addition, the load capacity factor rises by 0.32 to 0.35% for 1% increase in the usage of renewable energy. This finding demonstrates that the BRICS nations are now using enough renewable energy to improve the environment. On the other hand, new evidence continues to support the detrimental consequences of foreign capital investments on the environment in the BRICS nations. The load capacity factor is decreased by 0.1 to 0.3% for 1% increase in FDI inflow.

**Table 8:** Results from CCE-MG estimation

	Model I	Model II
gdp	− 2.774** [1.333]	− 2.971* [1.605]
gdp^2^	0.121* [0.071]	0.209* [0.112]
ren	0.353*** [0.067]	0.329*** [0.049]
fdi	− 0.034*** [0.062]	− 0.011** [0.006]
esi	0.041*** [0.018]	0.033*** [0.014]
esi*fdi	-	0.006*** [0.002]

^*^, **, and *** indicate statistical significance at 10, 5, and 1% level, respectively. Numbers in brackets are standard errors

The CCE-MG findings also support the environmental effectiveness of environmental policies in BRICS nations. The load capacity factor rises by 0.03 to 0.04% for 1% increase in the environmental stringency index. The association between foreign direct investments and environmental quality supports the long-term moderating impact of environmental rules.

### Discussions

**Fig. 1:**
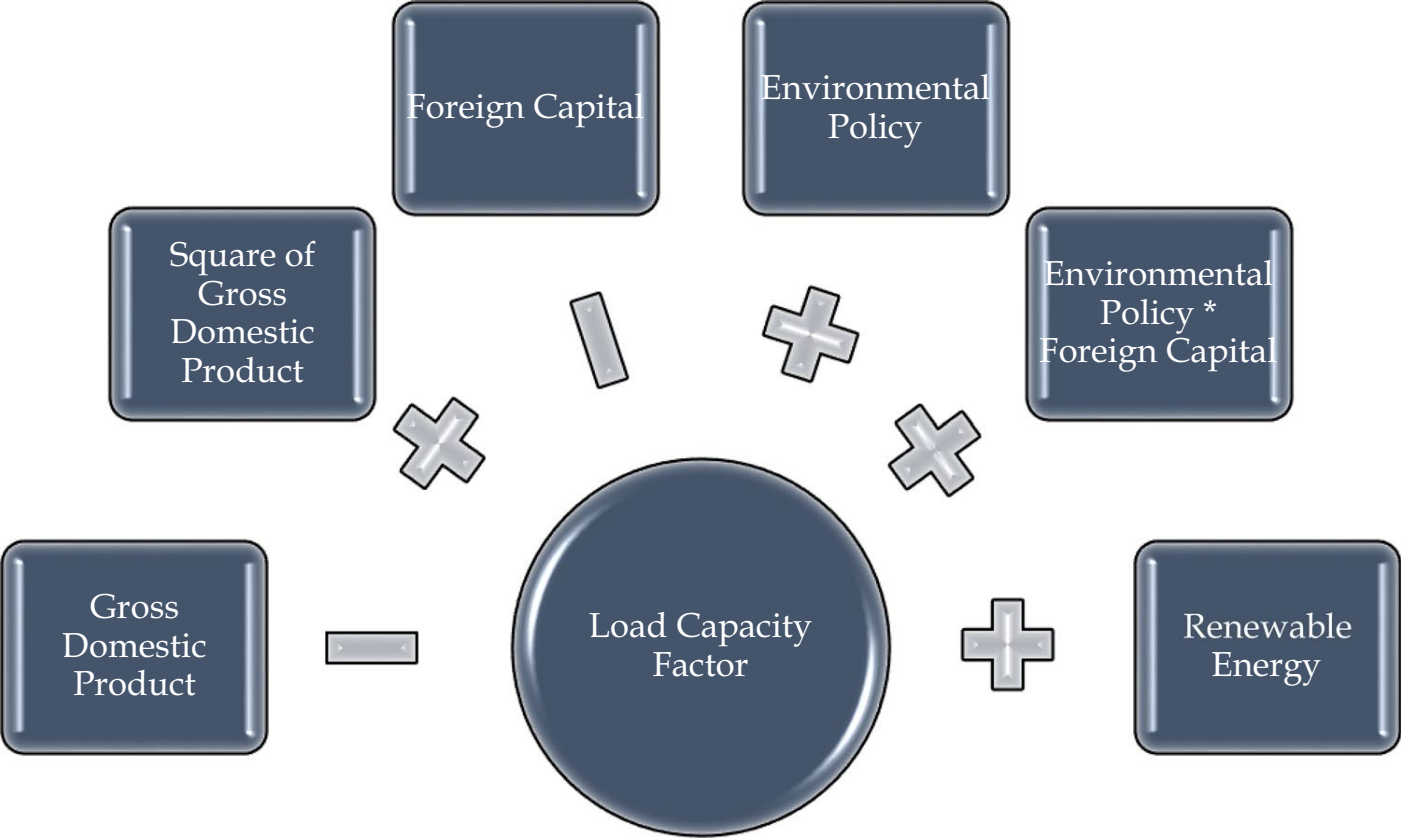
Summary of empirical findings

The empirical findings from whole empirical analyses are summarized in Fig. 1. We may first talk about the U-shaped link between economic growth and environmental quality after discussing the results from the empirical analyses and the potential causes for these findings in depth. This conclusion is also corroborated by Kartal et al. (2023), Jin et al. (2023), and Guloglu et al. (2023). Regarding the potential causes of this finding, nations often see a sharp rise in their ecological footprints during the early phases of economic growth and industrialization as a result of increasing resource use and pollution. The downward sloping component of the U-shaped curve results from a reduction in the load capacity factor as the environment attempts to deal with these stresses. On the other hand, when economies expand, they often diversify away from resource-intensive sectors and make investments in more efficient and environmentally friendly technology. This diversity may lessen the ecological footprint associated with each unit of economic production and help the curve’s upward slope as the load capacity factor rises. Additionally, technological and innovative developments may result in cleaner and more sustainable industrial processes. Early investments in environmentally friendly technology by developing nations may result in an earlier shift to an upward sloping curve. Increased consumer awareness could be another factor. Consumers may become more environmentally aware and demand environmentally friendly goods and services as their wages rise. Businesses could implement more environmentally friendly practices and lessen their ecological footprints because of this shift in customer expectations.

The second major conclusion is that the load capacity factor grows as the proportion of renewable energy in overall energy consumption rises. The following are some potential reasons of this observation, which is consistent with Destek and Pata (2023), Ullah et al. (2023), and Destek et al. (2023c). A nation’s reliance on fossil fuels, which is linked to high carbon emissions and environmental damage, may be decreased by increasing the percentage of renewable energy sources like wind, solar, and hydropower. Reduced use of fossil fuels may result in a smaller ecological impact and an increase in load capacity factor. In addition, compared to fossil fuels, renewable energy sources often emit less greenhouse gases. This decrease in emissions may have a favorable impact on load capacity, resulting in less water and air pollution, better air quality, and a healthier ecosystem. Additionally, as compared to infrastructure and fossil fuel extraction, renewable energy projects like wind and solar power plants often leave less of an ecological impact. They are less prone to damage natural ecosystems, preserving biodiversity and the health of the ecosystem and improving the load capacity factor.

The study’s key finding—which supports Solarin and Al-Mulali (2018) and Usman et al. (2022)—is that a growth in foreign direct investment causes environmental quality to decline. Investments in sectors like mining, agriculture, and manufacturing are often included in FDI, which may result in greater resource exploitation. By lowering the load capacity factor, these activities might contribute to environmental deterioration if they are not carried out sustainably and in accordance with the necessary environmental rules. Similar to how FDI may result in industrialization and greater output, which raises pollution and resource consumption, is the case with FDI. These activities may have a negative impact on the environment if there are insufficient regulations on the environment and enforcement. In fact, some FDI ventures could concentrate on sectors that use a lot of energy and fossil fuels. These businesses may increase carbon emissions and air pollution, which may have a negative impact on the environment. The fact that certain foreign investors should put short-term profit maximization above long-term environmental sustainability is another crucial factor. This may result in actions that endanger the environment in order to reap quick financial rewards.

Finally, we discover that for the BRICS nations, the negative impacts of foreign investments on environmental quality are reversed with the growth in environmental legislation. In fact, the interaction term’s (esi*fdi) favorable influence on the load capacity factor may suggest that investments made by foreign investors benefit the environment when they adhere to stringent environmental standards. This could include using more eco-friendly technology, sustainable habits, and pollution prevention techniques.

Foreign direct investments (FDI) from nations with cutting-edge environmental technology and practices may also improve the environment in host nations. With these investments, pollution and resource consumption might be reduced via the use of cutting-edge, environmentally friendly technology. In light of stringent environmental restrictions, some foreign investors may give priority to “green” or sustainable initiatives. Investments that might increase the load capacity factor could be made in renewable energy, energy efficiency, and environmentally friendly industrial techniques. In fact, nations with high “esi” ratings are more likely to attract foreign direct investment from businesses that are already committed to environmental sustainability. These investors could decide to fund ecologically beneficial initiatives that abide by the laws of the host nation. Additionally, beneficial connections between “esi” and “fdi” could be the result of policy synergies that are supported by stringent environmental rules, incentives, and support for sustainable foreign investment. The results of this mixture may be more favorable for the environment.

## Conclusions and policy implications

### Conclusions

The major goal of this research is to determine if environmental regulations have a moderating influence on the link between foreign direct investments and the load capacity factor for BRICS nations. A timeframe including the yearly data from 1992 to 2020 is examined for this purpose using second-generation panel data analysis. To prevent omitted variable error, real income per capita, the proportion of renewable energy use in total energy consumption, and the environmental stringency index are also included in the empirical model. To account for a potential parabolic link between economic development and environmental quality, the model also incorporates the square of real income as an independent variable. In order to examine the moderating effect of environmental laws, the model additionally includes the interaction terms of the environmental stringency index and foreign direct investments.

According to empirical data, economic growth and environmental quality have a long-term U-shaped connection. Additionally, by raising the load capacity factor, rising renewable energy consumption enhances environmental quality. On the other side, it is found that greater foreign direct investment inflow decreases the load capacity factor, which is why larger inflows of foreign capital are bad for the environmental quality of BRICS nations. The increased environmental rules show the load capacity factor’s environmental effectiveness by increasing it. Finally, it can be demonstrated that the parameter of the relationship between environmental regulation and foreign capital is positive. This suggests that the detrimental impacts of foreign direct investment on the environment are reversed by the tightening of environmental rules. In other words, environmental restrictions’ ability to moderate behavior has been established.

### Policy implications

For the established presence of the moderating function of environmental regulations in the link between foreign investment and environmental quality, the following policies might be recommended:

i) The BRICS nations should keep tightening their environmental rules to make sure that projects involving foreign direct investment adhere to high environmental standards. This comprises the creation and application of stringent laws, rules, and standards that deal with resource management, pollution control, and sustainable practices.
ii) Promoting foreign direct investment in accordance with sustainability objectives. Governments may reward foreign investors that emphasize environmentally friendly initiatives like renewable energy, energy efficiency, and green technology with incentives and special treatment.
iii) Projects involving foreign direct investment that make investments in environmentally friendly technology and practices should be given financial incentives, tax breaks, and subsidies. These incentives may influence international investors to use environmentally friendly practices.
iv) In order to make sure that international investors are aware of environmental standards and committed to abiding by them, governments may work with foreign investors. FDI aims and environmental targets may be more closely aligned via ongoing discussions, collaborations, and information exchange.
v) It should be remembered that depending on the industry, the effects of foreign direct investments on environmental sustainability may differ. To address the environmental issues and possibilities unique to individual sectors, policies and laws must be adapted.
vi) To guarantee effective enforcement and oversight of rigorous environmental legislation, investments should be made in strengthening the competence of regulatory organizations and enforcement procedures.
vii) It is important to aggressively promote the transfer of technology from foreign investors to the host nation. Make sure that initiatives involving foreign direct investment provide cutting-edge, eco-friendly technology that can boost the regional economy.
viii) It should be noted that while the moderating influence cannot be shown in the near term but may be demonstrated over the long term, it may take some time for the good environmental effects of stringent laws and sustainable foreign direct investment to manifest. Governments should have a long-term mindset and refrain from compromising environmental objectives in favor of immediate economic benefits.

In the end, this study has a number of limitations. To begin with, the study solely takes load capacity into account as an environmental indicator. The results of future research that take into account various environmental indicators (such as load capacity, ecological footprint, carbon emissions, etc.) can be compared. Furthermore, it solely addresses how environmental policy might mitigate the negative consequences of foreign capital on environmental quality. However, it is also possible to assess the efficacy of variables like technical advancement and financial development.

## Data Availability

The datasets analyzed during the current study are available from the corresponding author upon reasonable request.

## Abbreviations

APEC: Asia-Pacific Economic Cooperation
CCE-MG: Common Correlated Effects Mean Group
CSD: Cross-sectional dependency
DSUR: Dynamic seemingly unrelated regression
ECM: Error correction model
EPSI: Environmental policy stringency index
FDI: Foreign direct investment
GDP: Gross domestic product
MENA: Middle East and North Africa
NAFTA: North American Free Trade Agreement
OECD: Organisation for Economic Co-operation and Development
PHH: Pollution haven hypothesis
SEM: Spatial Error Model
SLM: Spatial Lag Model
NARDL: Nonlinear Autoregressive Distributed Lag
